# Fluorescein Hydrazide-Appended Metal–Organic Framework as a Chromogenic and Fluorogenic Chemosensor for Mercury Ions

**DOI:** 10.3390/molecules26195773

**Published:** 2021-09-23

**Authors:** Aasif Helal, Muhammed Naeem, Mohammed Fettouhi, Md. Hasan Zahir

**Affiliations:** 1 Center of Research Excellence in Nanotechnology (CENT), King Fahd University of Petroleum and Minerals (KFUPM), Dhahran 31261, Saudi Arabia; g201908410@kfupm.edu.sa; 2 Department of Chemistry, King Fahd University of Petroleum and Minerals, Dhahran 31261, Saudi Arabia; fettouhi@kfupm.edu.sa; 3 Interdisciplinary Research Center for Renewable Energy and Power Systems, King Fahd University of Petroleum and Minerals (KFUPM), Dhahran 31261, Saudi Arabia; hzahir@kfupm.edu.sa

**Keywords:** metal–organic framework, composite, fluorescein hydrazide, chromogenic, fluorogenic

## Abstract

In this work, we prepared a fluorescein hydrazide-appended Ni(MOF) (Metal–Organic Framework) [Ni_3_(BTC)_2_(H_2_O)_3_]·(DMF)_3_(H_2_O)_3_ composite, FH@Ni(MOF). This composite was well-characterized by PXRD (powder X-ray diffraction), FT-IR (Fourier transform infrared spectroscopy), N_2_ adsorption isotherm, TGA (thermogravimetric analysis), XPS (X-ray photoelectron spectroscopy), and FESEM (field emission scanning electron microscopy). This composite was then tested with different heavy metals and was found to act as a highly selective and sensitive optical sensor for the Hg^2+^ ion. It was found that the aqueous emulsion of this composite produces a new peak in absorption at 583 nm, with a chromogenic change to a pink color visible to the naked eye upon binding with Hg^2+^ ions. In emission, it enhances fluorescence with a fluorogenic change to green fluorescence upon complexation with the Hg^2+^ ion. The binding constant was found to be 9.4 × 10^5^ M^−1^, with a detection limit of 0.02 μM or 5 ppb. This sensor was also found to be reversible and could be used for seven consecutive cycles. It was also tested for Hg^2+^ ion detection in practical water samples from ground water, tap water, and drinking water.

## 1. Introduction

Mercury is considered to be a highly toxic heavy metal and non-biodegradable pollutant [1]. As an environmental pollutant, it exists in three primary forms in the environment: inorganic mercury (Hg^2+^), elemental mercury (Hg^0^), and organic mercury (such as methylmercury, ethylmercury, and phenylmercury) [2]. Among these, the most common and stable form of mercury is Hg^2+^. This is highly soluble in water and causes severe health problems [3,4]. The principal sources of this metal ion are natural, such as volcanic emissions, and anthropogenic, such as industrial waste from metal finishing, paint production, electronic devices, batteries, electroplating, mine drainage, and the metallurgical alloy industry [5]. The mercury ion is bio-accumulated throughout the food chain due to its water stability, non-degradability, and physiological toxicity. The noxious nature of Hg^2+^ is due to the high binding affinity for the amino (-NH_2_) and thiol (-SH) groups of proteins, which have antagonistic effects on the immune system, digestive system, chromosomes, kidney function, pulmonary system, and central nervous system [6,7,8,9]. The excessive accumulation of mercury can lead to further diseases, such as Minamata, Acrodynia, and several other gastrointestinal tract diseases [10]. Several analytical techniques are available for the detection of mercury, such as atomic absorption spectroscopy (AAS), inductively coupled plasma atomic emission spectrometry (ICP-AES), and inductively coupled plasma mass spectrometry (ICP-MS). However, these methods are expensive, involving complex instruments with well-established infrastructures, and require pervasive sample pretreatment that is laborious, time consuming, and associated with a high risk of contamination and sample loss. Thus, these methods are not suitable for in situ or instant analysis during field studies [11,12,13]. Several sensors based on organic compounds [14,15], nanoparticles [16,17], polymeric materials [18], proteins [19,20], magnetic nanocomposite s [21], and DNA-functionalized hydrogels [22,23] have been used for the detection of mercury. However, these materials have several disadvantages pertaining to their thermal and chemical stability, multistep synthesis, selectivity, and sensitivity.

Metal–Organic Frameworks are extended porous crystalline structures that have been used extensively by chemists and material engineers. Due to their crystalline nature, high porosity, tunable pores (microporous and mesoporous), and moderately high stability, they represent significant materials in the field of gas adsorption and separation [24], catalysis [25], photocatalysis [26], biological applications [27], electrochemical applications [28], and sensing [29]. Luminescent MOFs have been extensively used as fluorescent chemosensors for the detection of different analytes. The pervasive surface area and open channels in MOFs permit definitive analytes’ fast diffusion for highly sensitive fluorescence detection. These pores can be efficiently functionalized to cater for readily available interactional sites and confined environments for the highly selective detection of specific analytes [30,31]. Moreover, the crystalline nature of the MOF helps in the interpretation of the host–guest interaction and its photophysical nature [32].

MOFs have been extensively used in dye adsorption due to their modifiable porous structure [33,34,35,36]. Furthermore, dye-incorporated MOFs have often been used for white light emission [37], the detection of nitro explosives [38], metal ions [39], organic molecules [40], photocatalysis [41], two-photon-pumped lasing [42], and solar cell manufacturing [43]. The xanthene-based fluorescein dye has been most extensively used, as it has a bright signal with a high quantum yield and is nontoxic. In addition, excitation and emission wavelengths occur in the visible region of the spectrum, and these wavelengths have multiple reactive sites in their skeletons [44]. The functionalization of the carboxylic acid is the most common factor that results in the formation of spirolactam-based chemosensors, which, upon the ring opening, form highly emissive probes [45].

In this paper, we report the preparation of a composite of fluorescein hydrazide that has been coordinately bonded to the nickel SBU of a nickel-based MOF [Ni(MOF)] [Ni_3_(BTC)_2_(H_2_O)_3_]·(DMF)_3_(H_2_O)_3_ [46]. This composite was then used for the optical detection of heavy metals. The composite was well-characterized by PXRD, nitrogen adsorption isotherm, FTIR, TGA, FESEM, and XPS. We also studied its photophysical properties, which indicated that it could act as a chromogenic and fluorogenic sensor for the detection of mercury.

## 2. Results and Discussions

### 2.1. Synthesis and Characterization of FH@Ni(MOF)

**Scheme 1:**
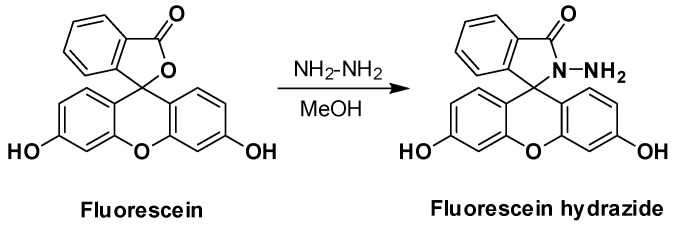
Synthesis of fluorescein hydrazide (FH).

**Scheme 2:**
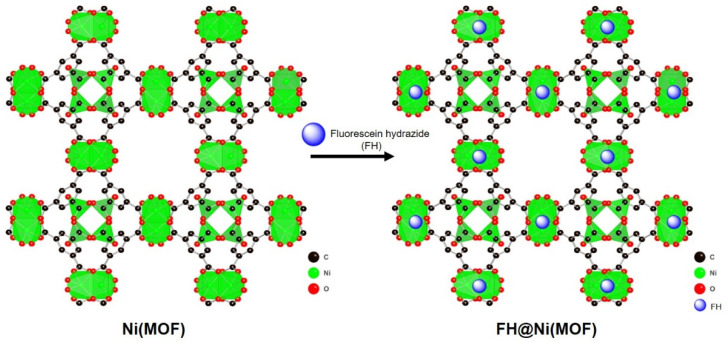
Synthesis of FH@Ni(MOF) from Ni(MOF).

The fluorescein hydrazide was synthesized using the methods given in the literature and characterized by the ^1^H and ^13^C NMR (Scheme 1) (Figures S1 and S2). The Ni(MOF) was prepared according to the method followed in the literature [46]. Then, it was activated by evacuation at a high temperature to de-coordinate the aqua ligands from the nickel clusters and generate coordinately unsaturated sites (CUS). Upon interaction with the fluorescein hydrazide, the CUS are coordinately occupied with the lone pair electrons of the nitrogen in fluorescein hydrazide to give the composite FH@Ni(MOF) (Scheme 2).

**Figure 1:**
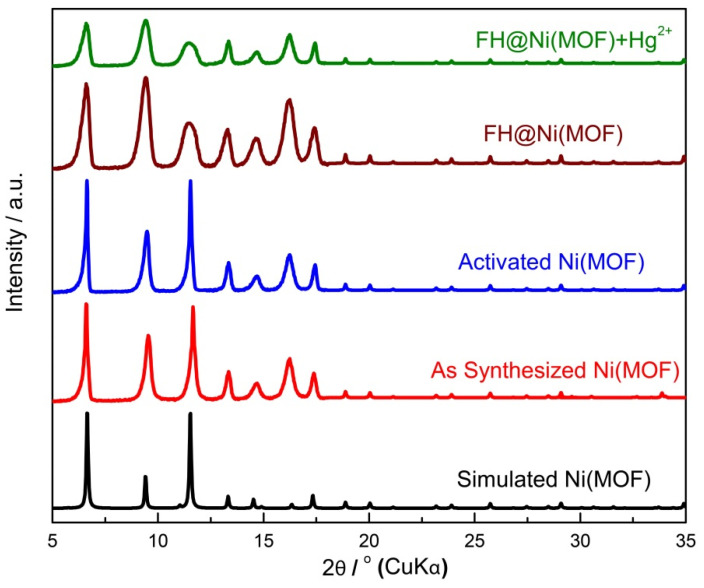
Powder X-ray diffraction (PXRD) analysis of FH@Ni(MOF). The simulated pattern (black) of Ni(MOF) matches well with the as-synthesized sample of Ni(MOF) (red), activated Ni(MOF) (blue), FH-appended sample FH@Ni(MOF) (brown), and FH@Ni(MOF) after the addition of Hg^2+^ (green).

**Figure 2:**
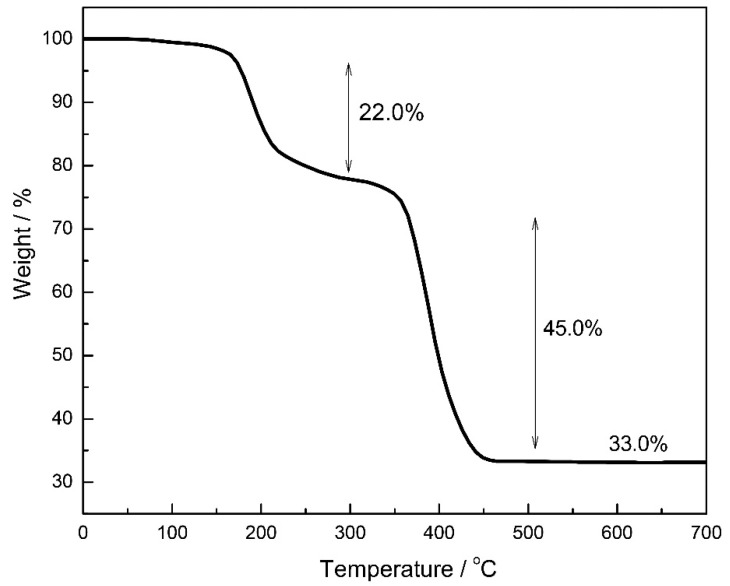
Thermogravimetric analysis of FH@Ni(MOF).

**Figure 3:**
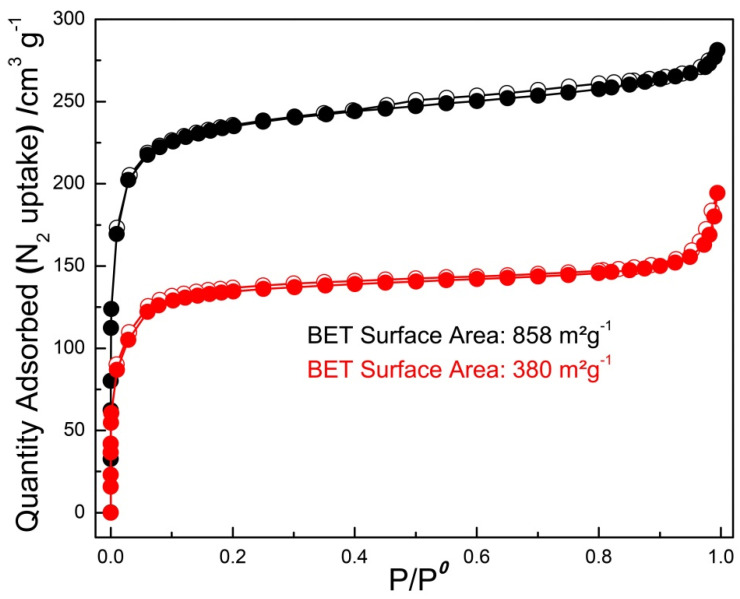
N_2_ adsorption isotherm of Ni(MOF) and FH@Ni(MOF).

The structural characterization of the composite was carried out using the PXRD, which confirmed that the consistency in the crystallinity and phase purity was well retained in the as-synthesized Ni(MOF), activated Ni(MOF), and FH-appended FH@Ni(MOF) (Figure 1). In the FTIR spectrum (Figure S3) of the FH@Ni(MOF), the bands at 701 cm^−1^ and 754 cm^−1^ correspond to the out-of-plane aromatic C-H bending modes of the benzene ring of the linker, while the band at 936 cm^−1^ is due to the bending mode of the aromatic C-H of the fluorescein hydrazide. The band at 1111 cm^−1^ is designated to the C-H in-plane bending of the benzene ring, while the band at 1185 cm^−1^ is assigned to the fluorescein hydrazide’s N-N stretching. The symmetric and asymmetric stretching modes of the carbonyl moiety in the COO^−^ group are represented by the strong bands at 1378 and 1439 cm^−1^ and 1579 and 1629 cm^−1^, respectively. The bands at 1499 cm^−1^ and 1680 cm^−1^ are likely due to the in-plane bending of the H-N-N and C=O stretching of the fluorescein hydrazide, respectively. The small band at the 3415 cm^−1^ corresponds to the O-H stretching vibration of the fluorescein hydrazide. The simultaneous presence of both the IR bands from the Ni(MOF) and fluorescein hydrazide in FH@Ni(MOF) illustrates the formation of the composite. In order to study the thermal stability of the composite FH@Ni(MOF), we carried out a thermogravimetric analysis. From Figure 2, it can be observed that weight loss occurred in two stages: (a) In the first step, there was a weight loss of 22.0% at 150 °C, which corresponds to the decomposition of the fluorescein hydrazide. (b) The second step showed a sudden weight loss of about 45.0% at 375–450 °C due to the thermal disintegration of the framework. The final silt of 33.0% can be ascribed to the nickel oxide formed after decomposition. The N_2_ adsorption isotherm of the FH@Ni(MOF) indicates that it is microporous, with a characteristic Type I isotherm (Figure 3). The Brunauer–Emmett–Teller surface area was calculated to be 380 m^2^/g. The surface area of FH@Ni(MOF) was much abated compared to the pristine Ni(MOF) due to the presence of the fluorescein hydrazide occupying the pores of the MOF. The XPS (Figure S4) spectra of Ni(MOF) and FH@Ni(MOF) were recorded; both the spectra had peaks at 856.1 eV and 873.3 eV, corresponding to Ni 2p^3/2^ and 2p^1/2^, indicating that nickel mainly exists as Ni^2+^. The peak at 397.2 eV is also attributed to Ni 1s. In both the spectra, the peaks at 284.1 eV (C 1s) and 531.9 eV (O 1s) resemble the characteristic peaks for C and O, respectively. These peaks’ intensities in the case of FH@Ni(MOF) are much higher than those for the pristine Ni(MOF), indicating the presence of fluorescein hydrazide. Furthermore, the presence of an additional peak at 401.6 eV in the case of FH@Ni(MOF) indicates the presence of N 1s from the fluorescein hydrazide. The scanning electron microscopy (SEM) images of the microcrystalline composite FH@Ni(MOF) show a uniform morphology of rod-shaped structures assembled into sheets (Figure S5). 

The amount of fluorescein hydrazide appended in the FH@Ni(MOF), as calculated by the alkaline digestion, was found to be approximately 0.211 g g^−1^ (0.61 mmole.g^−1^) of FH@Ni(MOF).

### 2.2. Cation Sensing Properties of FH@Ni(MOF)

**Figure 4:**
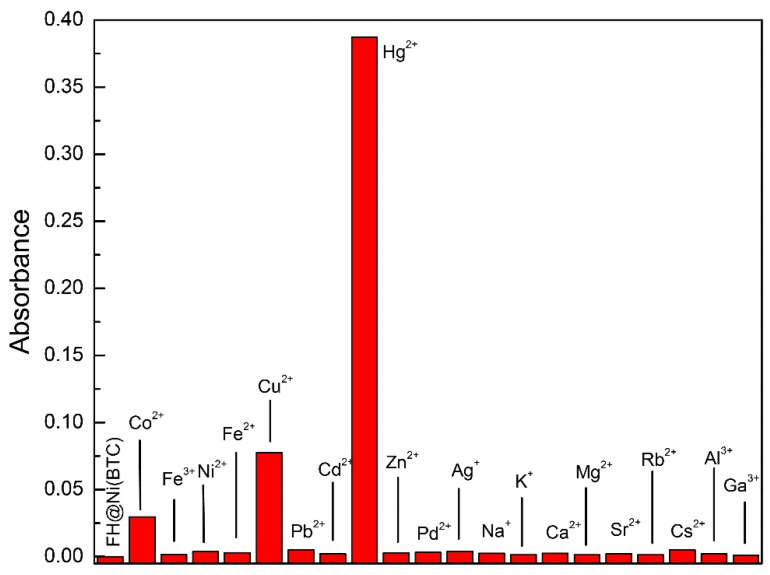
Change in the UV–vis absorbance of FH@Ni(MOF) in water upon addition of 200 μL of different metal cations (10^−2^ M).

**Figure 5:**
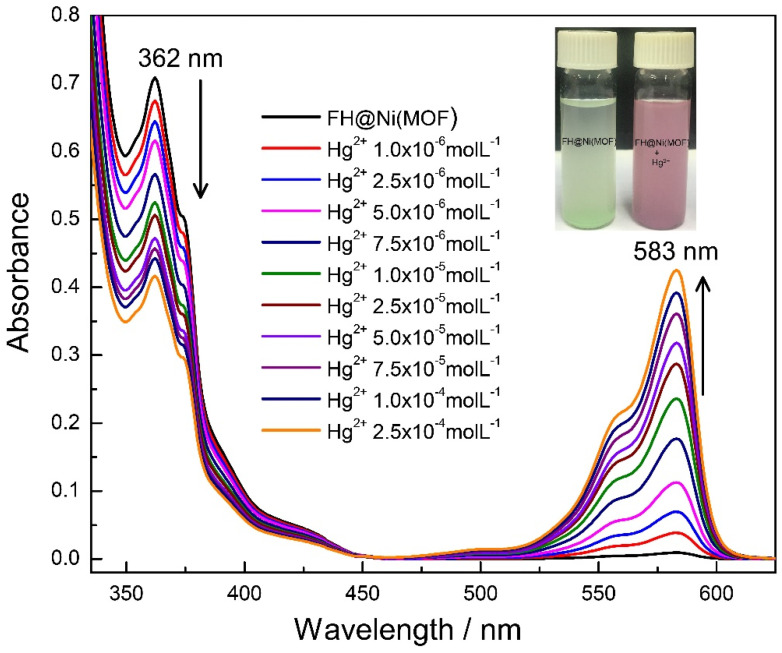
Changes in the UV–vis spectra of FH@Ni(MOF) with the incremental addition of Hg^2+^ (10^−2^ M) in water. Inset: change in the color of FH@Ni(MOF) upon the addition of Hg^2+^.

Ni(MOF) does not have effective optical or binding properties for heavy metals. Nevertheless, it produces both optical and selective binding properties with heavy metals when forming a composite with fluorescein hydrazide. Thus, the absorbance and emission studies of FH@Ni(MOF) were carried out in an aqueous solution such as an emulsion. The FH@Ni(MOF) has an absorption maximum at 362 nm due to the π−π* transition of the aromatic rings. Initial studies with different metal cations indicate that only the Hg^2+^ ion produces a decrease in the absorbance at 362 nm and the appearance of a new absorption maximum at 583 nm (Figure 4 and Figure S6). This peak is characteristic of the opening of the spirolactam ring and binding with the Hg^2+^ (Scheme 2). This new absorption triggers the color change of the emulsion from colorless to pink (Figure 5), which is visible to the naked eye. The slow addition of Hg^2+^ to the emulsion of FH@Ni(MOF) resulted in the formation of the new peak at 583 nm, with a synchronous decrease in the absorbance band at 362 nm (Figure 5). From the UV–vis titration, the binding constant was calculated to be 6.1 × 10^5^ M^−1^ (error estimated to be ≤10%) (Section S3 and Figure S10) (Supplementary Maerials). Moreover, the Job’s plot experiment of FH@Ni(MOF) with the Hg^2+^ ion indicated the formation of a 1:1 complex between the fluorescein hydrazide of the FH@Ni(MOF) and the Hg^2+^ ion (Figure S7).

**Figure 6:**
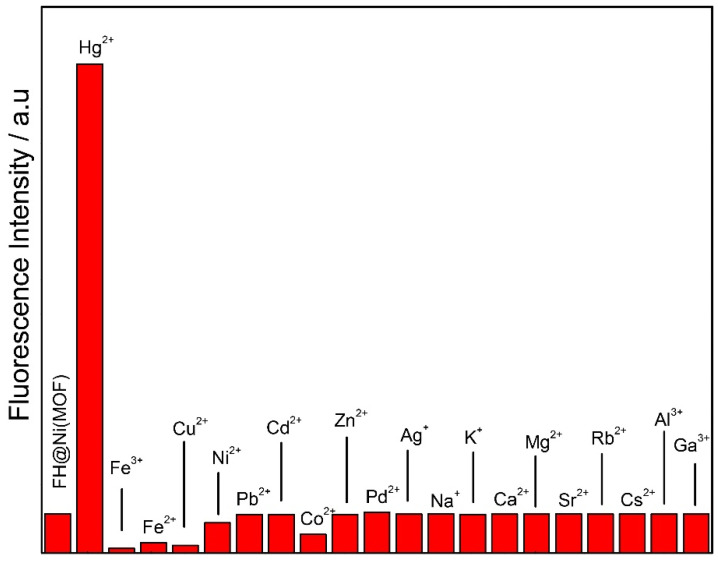
Change in the fluorescence intensity of FH@Ni(MOF) in water upon the addition of 200 μL of different metal cations (10^−2^ M).

**Figure 7:**
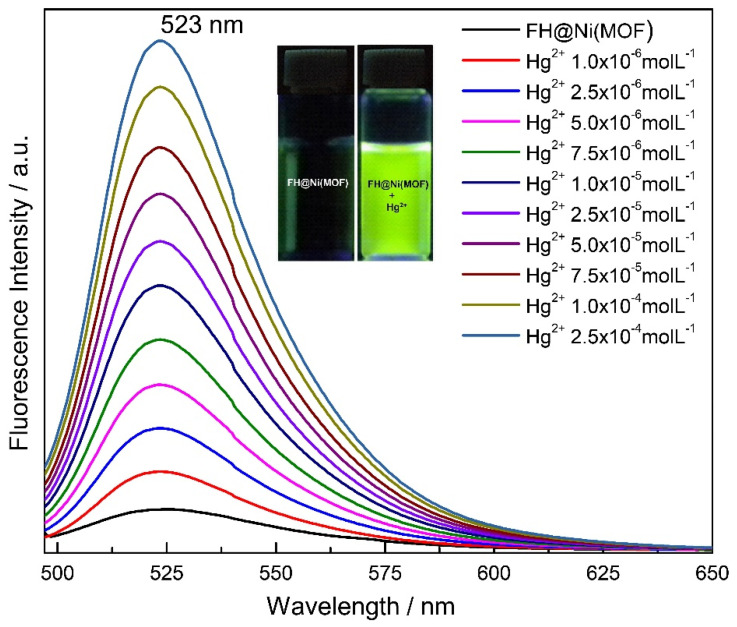
Changes in fluorescence emission spectra of FH@Ni(MOF) with the incremental addition of Hg^2+^ (10^−2^ M) in water (λ_ex_ = 460 nm). Inset: fluorogenic changes in FH@Ni(MOF) upon the addition of Hg^2+^ in water upon illumination at 365 nm.

The emission properties of composite FH@Ni(MOF) were investigated in aqueous emulsion with different biologically and non-biologically relevant cations. It was observed that only Hg^2+^, in addition to the composite FH@Ni(MOF), produced an enhancement in the emission at 523 nm upon excitation at 460 nm (Figure 6). None of the cations, except Hg^2+^, induced any noticeable enhancement in emission when interacting with any metal ions. However, transition metal ions Cu^2+^, Co^2+^, and Fe^3+^ induced complete or partial quenching on binding with the chemosensor due to their paramagnetic nature (Figure S8). This high selectivity is likely due to the attachment of the fluorescein hydrazide with the inorganic SBU (secondary building unit) of the Ni(MOF) in the composite FH@Ni(MOF). Upon the slow addition of Hg^2+^ to FH@Ni(MOF), we detected the enhancement of the peak at 523 nm upon excitation at 460 nm (Figure 7) with the change in the color of the emulsion to green fluorescence (λ_ex_ = 365 nm) (Figure 7). The augmentation of the peak at 523 nm was linear with the increase in the concentration of the Hg^2+^. To quantify the complexation nature between the Hg^2+^ and FH@Ni(MOF), the Job’s plot analysis, in fluorescence, was executed by changing the sensor–cation concentration ratio. The maximum emission in the Job’s plot occurred at the mole fraction of 0.5 or 1:1 metal to metal–MOF complex (Figure S9). The quantum yield calculated from the integrated sphere, before and after the Hg^2+^ ion binding, was found to be increased from 0.07 to 0.46. This indicates that the complexation of Hg^2+^ with the FH@Ni(MOF) increases the charge transfer character of the composite, inhibiting the major nonradiative decay pathway. The binding constant between the metal (Hg^2+^) and the MOF composite (FH@Ni(MOF) obtained from the fluorescence titration was calculated to be 9.4 × 10^5^ M^−1^ (error estimated to be ≤10%) (Figure 7, Section S3, and Figure S11) [47]. The detection limit for Hg^2+^ by this sensor was calculated to be 0.02 μM or 20 nM (5 ppb) (Section S4 and Figure S12) [48]. This was found to be lower than the guidelines set by the World Health Organization (WHO) and the United States Environmental Protection Agency (US-EPA) for a maximum contaminant level of Hg^2+^ in drinking water of 2–6 mg/L (10 nM to 30 nM) [49,50]. The powdered XRD of the Hg^2+^-bounded FH@Ni(MOF) indicates that the crystallinity of the MOF is intact (Figure 1).

To delve into the possibility of using FH@Ni(MOF) for practical purposes, competitive binding experiments with 200 μL of various metal ions (10^−2^ M) in the presence of 200 μL of the Hg^2+^ ion exhibited that there was no interference by any of the metal ions in the enhancement of the emission of FH@Ni(MOF) by Hg^2+^ (Figure S13).

**Scheme 3:**
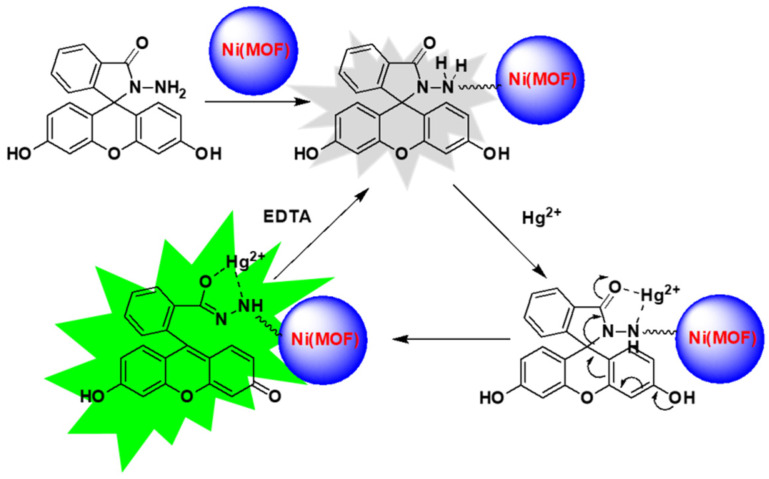
Schematic illustration of the mechanism for the detection of Hg^2+^ by FH@Ni(MOF).

The mechanism of fluorescence can be demonstrated based on Scheme 3. It can be clearly observed that the binding of the Hg^2+^ ion with the fluorescein hydrazide leads to the opening of the spirolactam ring, which produces a chromogenic change to a pink color that is visible to the naked eye. Moreover, the binding of the Hg^2+^ ion with the fluorescein hydrazide produces the chelation enhancement of fluorescence (CHEF), resulting in the enhancement of fluorescence at 523 nm upon excitation at 460 nm. We also checked the UV–Vis absorbance of the Ni(MOF) and FH with different metal ions in water and found that this selective chromogenic change in absorbance is only produced by the FH@Ni(MOF) upon binding with the Hg^2+^ ion (Figures S14–S16).

In order to measure the recyclable sensing ability of FH@Ni(MOF), the fluorescence- sensing experiments were repeated with the recovered materials. The first set of experiments was followed by washing with a 1.0 M aqueous solution of EDTA (ethylenediaminetetraacetic acid) in order to remove the bounded Hg^2+^ ions, water, and then drying at 100 °C for 1 h. The recovered FH@Ni(MOF) exhibited no significant change in emission intensity or sensitivity towards detecting Hg^2+^ ions for seven successive cycles (Figure S17). We also used FH@Ni(MOF) to detect Hg^2+^ ion in tap water, drinking water, and groundwater using the standard addition method [51]. From Table 1, the recovery yield of Hg^2+^ ions ranging from 97–101% indicates the efficacy of FH@Ni(MOF) for the detection of Hg^2+^ ions.

## 3. Experimental

### 3.1. Materials and General Methods

#### 3.1.1. Chemicals Used in This Work

Trimesic acid (BTC) (95%), nickel nitrate hexahydrate (99.9% purity), fluorescein (99.9% purity), *N*,*N*-dimethylformamide (DMF; 99.8% purity), ethanol (99.9% purity), dichloromethane (99.8% extra dry grade), and all other nitrates or chlorides of the metal salts were purchased from Sigma Aldrich Corporation, USA. NMR solvents: dimethyl sulfoxide-*d*_6_ (DMSO-*d*_6_; 99.9% purity) was purchased from Cambridge Isotope. All chemicals were used without further purification. The water used in this work was double-distilled and filtered through a Millipore membrane. The solutions of metal ions were prepared from their nitrate and chloride salts, and anions were prepared from their sodium and potassium salts (analytical grade), followed by subsequent dilution to prepare the working solutions.

#### 3.1.2. Instrumentation

Powdered X-ray diffraction patterns of the samples were recorded using a Rigaku MiniFlex diffractometer, which was equipped with Cu-Kα radiation. The data were acquired over the 2θ range of 5° and 30°. The FT-IR spectra of FH@Ni(MOF) were obtained using a Nicolet 6700 Thermo Scientific, USA instrument in the range of 400–4000 cm^−1^ using KBr. Thermogravimetric analysis (TGA) of the samples were performed using a TA Q500, USA. In this study, an activated sample of FH@Ni(MOF) (10 mg) was heated in an alumina pan under airflow (60 mL min^−1^) with a gradient of 10 °C min^−1^ in the temperature range of 30–800 °C. The N_2_ adsorption isotherm of the MOFs for the BET surface area was calculated using a Micromeritics ASAP 2020 instrument, USA. The surface morphology of these materials were discerned using a field emission-scanning electron microscope (FESEM, LYRA 3 Dual Beam, Tescan, USA), which operated at 30 kV. The FESEM samples were prepared from suspension in ethanol. The absorption spectra of the MOF were studied using a Jasco V-670 spectrophotometer. Fluorescence spectra were measured using a Jasco, Spectrophotometer FP-8500, Japan equipped with a xenon discharge lamp and 1 cm quartz cells with a slit width of 2 nm for both the source and the detector. Quantum yield studies were carried out using a Fluoromax-4 equipped with Quanta-Phi integration sphere (Horiba), using a liquid sample holder at room temperature.

#### 3.1.3. Sample Preparation for Photophysical Studies

In a typical luminescence-sensing experimental setup, 1.0 mg of FH@Ni(MOF) powder was dispersed in 1 mL of water. A volume of 3 mL of dispersed aqueous solution of FH@Ni(MOF) was placed in a 1 cm quartz cuvette, and the absorption and emission responses were measured in situ after the incremental addition of freshly prepared analyte solutions. The mixtures were sonicated for 5 min after each incremental addition of the analytes for uniform dispersion during the luminescent measurements. All of the measurements were performed at 298 K.

#### 3.1.4. Practical Application in Water Samples

FH@Ni(MOF) was used for the detection of Hg^2+^ in tap water, drinking water, and ground water via the standard addition method. All the water samples were filtered three times through a 0.2 mm membrane filter. Then, these three water samples were spiked with 10 and 15 μM of Hg^2+^ and titrated against the FH@Ni(MOF).

### 3.2. Synthesis

#### 3.2.1. Synthesis of Fluorescein Hydrazide (FH)

Fluorescein (500 mg, 1.44 mmol) was dissolved in 20 mL methanol and to it was added an excess amount of hydrazine hydrate (0.25 mL, 5.05 mmol). The reaction mixture was refluxed for 4 h and then cooled to room temperature, poured into distilled water, and extracted with ethyl acetate (6 × 25 mL). The combined extract was washed with brine, dried with anhydrous sodium sulphate, filtered, and then concentrated under a reduced pressure to yield (71%) FH [52,53]. ^1^H NMR (400 MHz, DMSO-*d_6_*), δ (ppm): 6.47–6.39 (m, 4H), 6.60 (d, *J*= 3.0 Hz, 2H), 7.00–6.98 (m, 1H), 7.49 (t, *J*= 4.0 Hz, 2H), 7.79–7.77 (m, 1H), 9.84 (s, 2H). ^13^C NMR (100 MHz, DMSO-*d_6_*), δ (ppm): 65.12, 102.82, 110.43, 112.48, 122.84, 123.89, 128.43, 128.89, 129.78, 133.10, 152.00, 152.88, 158.66, 165.99 (Figures S1 and S2).

#### 3.2.2. Synthesis of Ni(MOF)

The Ni(MOF) [Ni_3_(BTC)_2_(H_2_O)_3_]·(DMF)_3_(H_2_O)_3_ was prepared by the same method as that reported in the literature [46]. Ni-MOF was synthesized by dissolving Ni(NO_3_)_2._ 6H_2_O (291 mg, 1.0 mmol), and Trimesic acid (BTC) (210 mg, 1.0 mmol) in DMF (20 mL) with ultrasonic vibration for 15 min, then 5 mL of acetic acid was added. The as-obtained mixture was transferred to a 40 mL Parr steel autoclave and heated at 448 K for 72 h. Then, the autoclave was cooled in air to room temperature. The resulting green icosahedral-shaped crystals were collected and washed with 3 × 10 mL of DMF for 3 days and 3 × 10 mL of CH_2_Cl_2_ for 3 days, yielding the required Ni-MOF in a 35% yield (related to the nickel salt).

#### 3.2.3. Synthesis of FH@Ni(MOF)

The Ni(MOF) was activated by heating the MOF at 150 °C in a vacuum oven for 6 h. The activated MOF (100 mg) was then suspended in ethanol (10 mL) containing fluorescein hydrazide (FH) (200 mg, 0.55 mmol), and refluxed at 358 K for 48 h. Then, the vial was cooled in air to room temperature. The resulting FH@Ni(MOF) was washed three times with DMF (5–10 mL) using a centrifuge (10,000 rpm for 30 min) and then sequentially immersed in methanol (5–10 mL three times per day) for three 24 h periods. The washing with methanol was continued until the washing solution did not contain any residual dye, which was confirmed by absorption studies of the methanol extracts obtained after washing. This justified the fact that no dye was leaching from the FH@Ni(MOF) powder. Finally, FH@Ni(MOF) was dried by removing the solvent under vacuum for 24 h at 80 °C. FT-IR (KBr, cm^−1^): 3415, 1680, 1629, 1579, 1499, 1439, 1378, 1185, 1111, 936, 836, 795, 754, 701. Anal. Calcd for C_144_H_108_N_12_O_33_Ni_3_[Ni_3_(BTC)_2_(H_2_O)_3_]. (C_21_H_16_N_2_O_3_)_6_: C 63.81; H 4.02; N 6.20; Found: C, 62.78; H, 4.15; N, 6.37.

## 4. Conclusions

In conclusion, we prepared a composite FH@Ni(MOF) by appending fluorescein hydrazide with the inorganic SBU of Ni(MOF). The composite was characterized by PXRD, FTIR, XPS, N_2_ adsorption isotherm, and TGA. This composite was found to act as a highly selective and sensitive optical sensor for Hg^2+^ ions and could also detect Hg^2+^ ions in the presence of other metal ions. It was observed that this composite produces a pink color visible to the naked eye and a green fluorescence upon binding with only the Hg^2+^ ion; none of the other metal ions produce such a chromogenic or fluorogenic change with this sensor. The binding constant was found to be 9.4 × 10^5^ M^−1^, with a detection limit of 0.02 μM, or 5 ppb. This chemosensor was also found to be reversible and could be used for seven consecutive cycles.

## Figures and Tables

**Table 1:** Determination of Hg^2+^ ions in water samples.

Sample	Hg^2+^ Ion (M) Spiked	Hg^2+^ Ion (M) Detected	Recovery (%)
Tap Water	10	10.09	100.9
	15	14.69	97.93
Drinking Water	10	10.1	101.0
	15	14.58	97.2
Groundwater	10	9.71	97.1
	15	14.71	98.07

FH@Ni(MOF) was used for the detection of Hg^2+^ by the standard addition method. All the water samples were filtered three times through a 0.2 mm membrane filter.

## Data Availability

Not applicable.

## Supplementary Materials

The following are available online. Section S1: Characterization of FH@Ni(MOF); Section S2: Optical Sensing properties of FH@Ni(MOF); Section S3: Determination of the Rate Constant; Section S4: Determination of the detection limit; Section S5: Comparison of Absorbance of FH, Ni(MOF), and FH@Ni(MOF) with metal ions; Section S6: Recyclability of FH@Ni(MOF); Section S7: References.

## Sample Availability

Not available as further studies on other applications are going on.
