# Supplementary material for: Fluorescein Hydrazide-Appended Metal–Organic Framework as a Chromogenic and Fluorogenic Chemosensor for Mercury Ions
Source: Molecules. 2021 Sep 23;26(19):5773. doi: 10.3390/molecules26195773 (PMC8510309; doi:10.3390/molecules26195773)
Supplement: Supplementary file 1 [file molecules-26-05773-s001.zip › molecules-1385681-supplementary.pdf]

Supplementary Material

Fluorescein Hydrazide Appended Metal-Organic Framework as Chromogenic and Fluorogenic Chemosensor for Mercury Ion

Aasif Helal,*[1] Muhammed Naeem,[1] Mohammed Fettouhi,[2] Md. Hasan Zahir[3],

[1]Center of Research Excellence in Nanotechnology (CENT), [2]Department of Chemistry, [3]Interdisciplinary Research Center for Renewable Energy and Power Systems, King Fahd University of Petroleum and Minerals (KFUPM), Dhahran 31261, Saudi Arabia

Table of Contents

**Section S1**	*Characterization of FH@Ni(MOF)*	**S2-S4**
**Section S2**	*Optical Sensing properties of FH@Ni(MOF)*	**S5-S6**
**Section S3**	*Determination of the Rate Constant*	**S7-S8**
**Section S4**	*Determination of the detection limit*	**S9-S10**
**Section S5**	*Comparison of Absorbance of FH, Ni(MOF), and FH@Ni(MOF) with metal ions*	**S11**
**Section S6**	*Recyclability of FH@Ni(MOF)*	**S13**
**Section S7**	*References*	**S13**

Section S1: **Characterization of FH@Ni(MOF)**

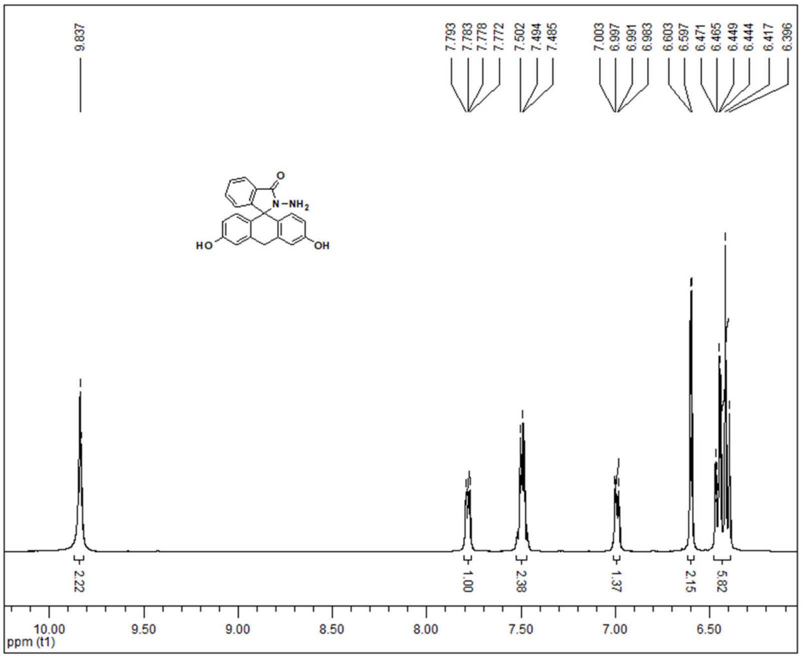

Figure S1. ^{1}H NMR spectrum of FH in DMSO-d_6 solution at 400 MHz.

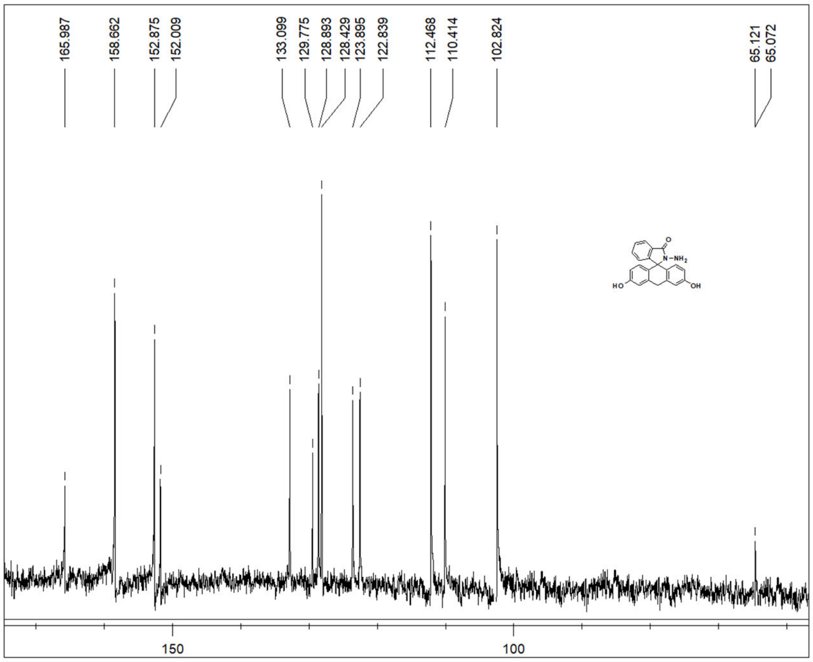

Figure S2. ^{13}C NMR spectrum of FH in DMSO-d_6 solution at 200 MHz.

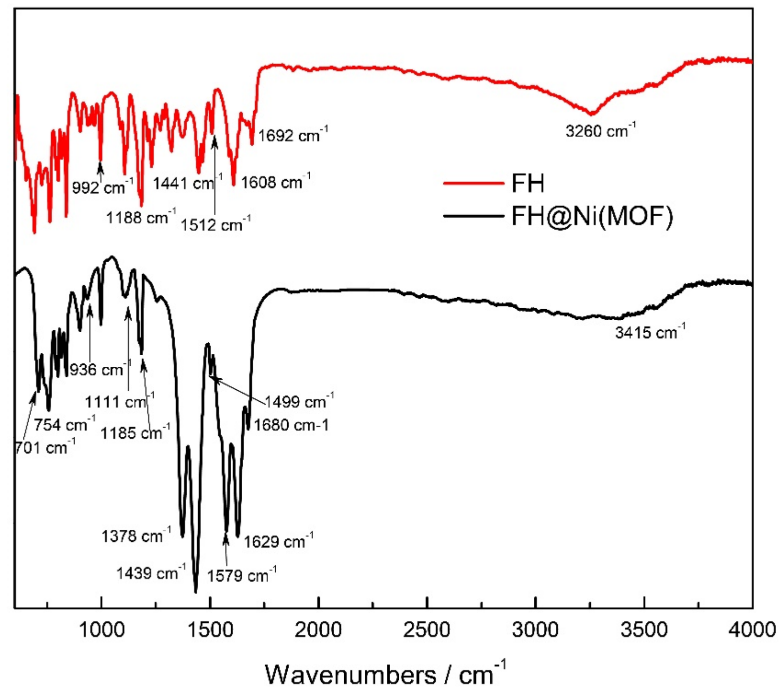

Figure S3: FTIR spectrum of FH and FH@Ni(MOF)

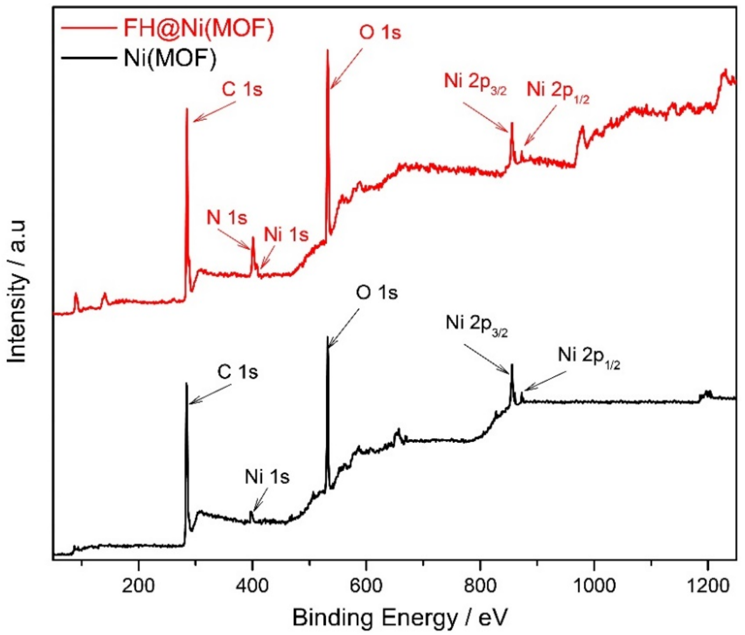

Figure S4: XPS spectrum of FH and FH@Ni(MOF)

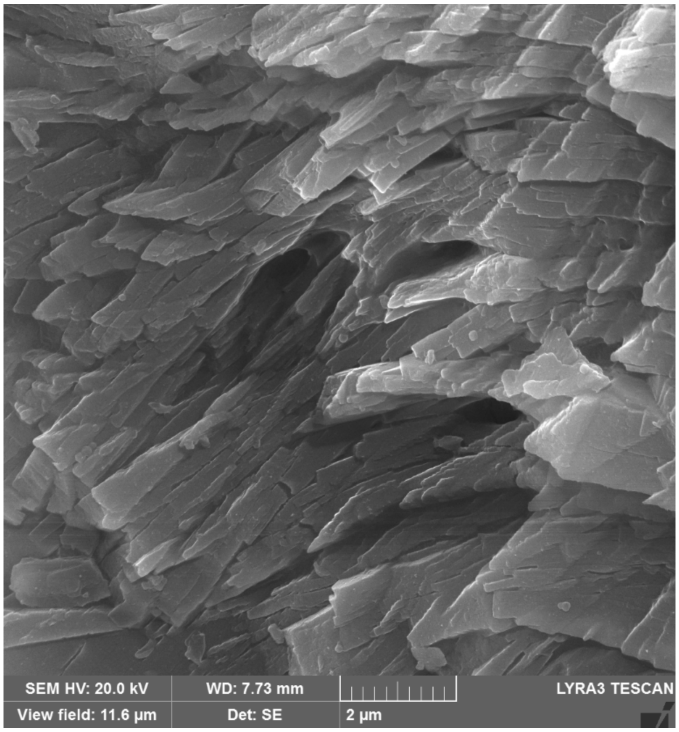

Figure S5: SEM of FH@Ni(MOF)

Section S2: Optical Sensing properties of FH@Ni(MOF)

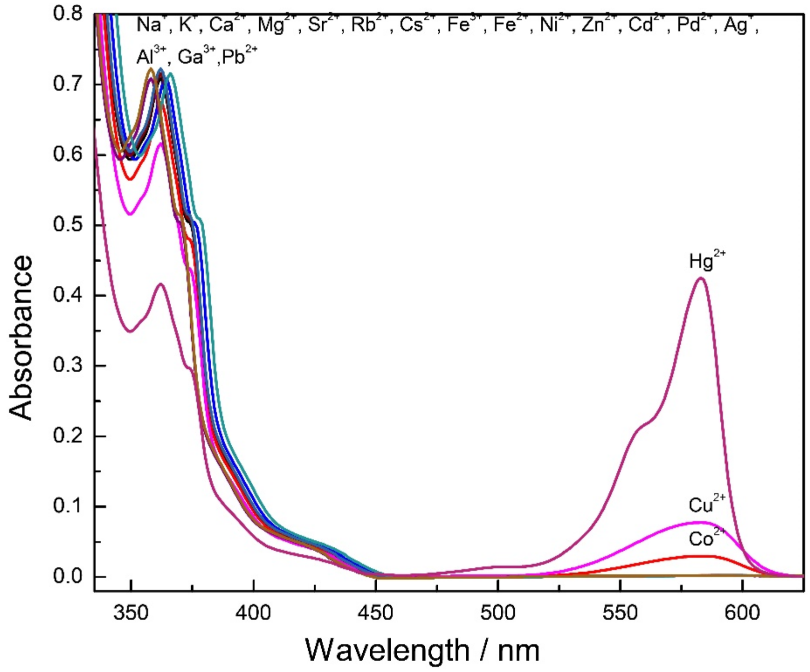

Figure S6. Change in the UV-vis spectrum of FH@Ni(MOF) in water upon addition of 200 μL of different metal cations (10^{-2} M).

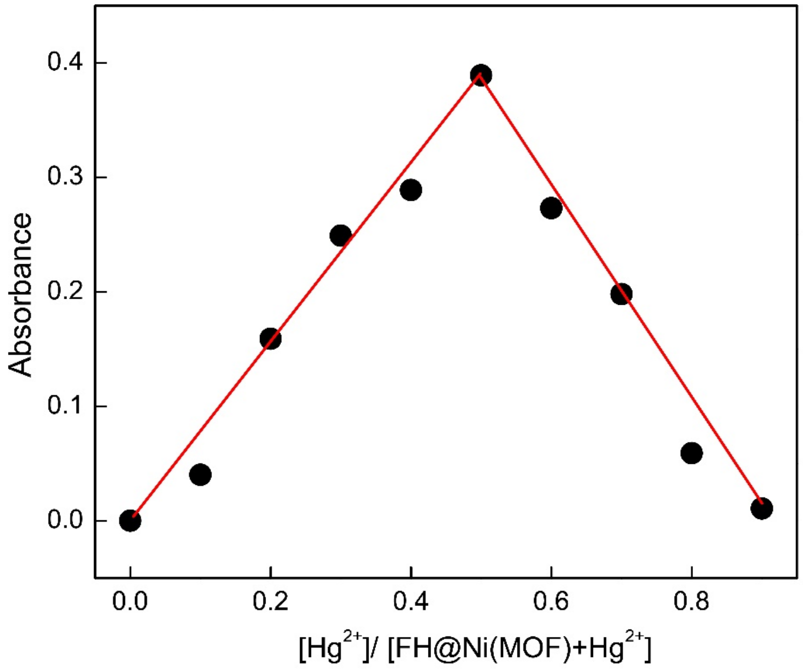

Figure S7. Job's plot for FH@Ni(MOF) with Hg^{2+} in water.

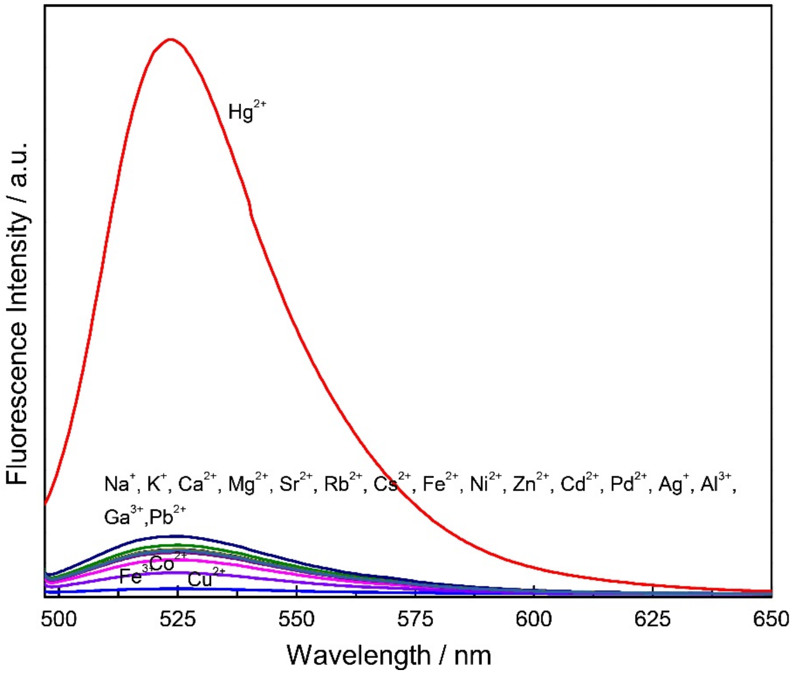

Figure S8. Change in the fluorescence emission spectrum of FH@Ni(MOF) in water upon addition of 200 μL of different metal cations (10^{-2} M).

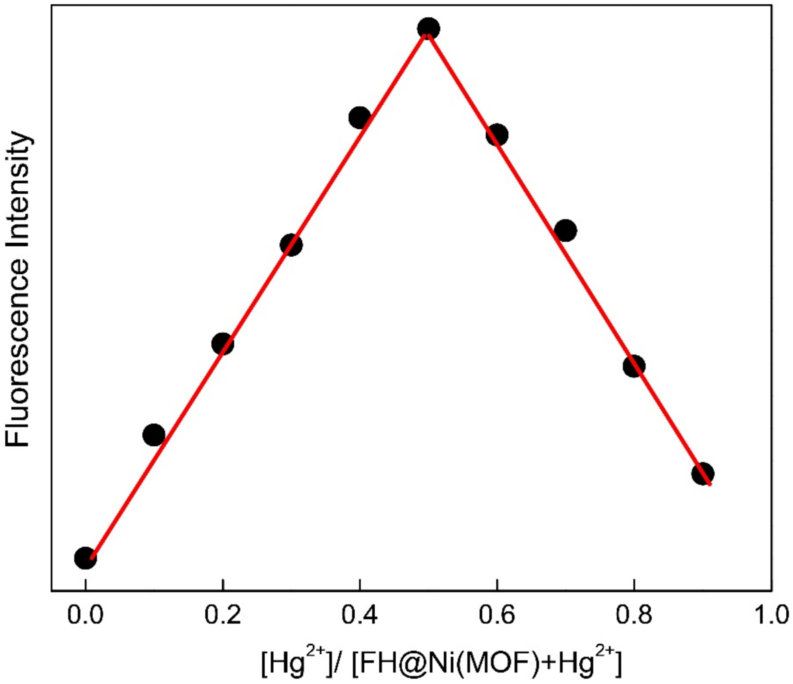

Figure S9. Job's plot for FH@Ni(MOF) with Hg^{2+} in water.

Section S3: **Determination of the Rate Constant:**

The rate constant was calculated using the following equation.[1]

$F_o/(F- F_o) = [a/(b - a)][(l/Ks[M]) + 1]$.

Where F_o is the absorbance or fluorescence intensities of the free host (here FH@Ni(MOF)), F is the absorbance or fluorescence intensities of the host-guest complex, a and b are constants, K_s is the rate constant and M is the concentration of the guest (here Hg^{2+}). When $F_o/(F- F_o)$ is plotted against the reciprocal of the concentration of the guest (here hydrazine) $[M]^{-1}$, the rate constant is given by the ratio intercept/slope.

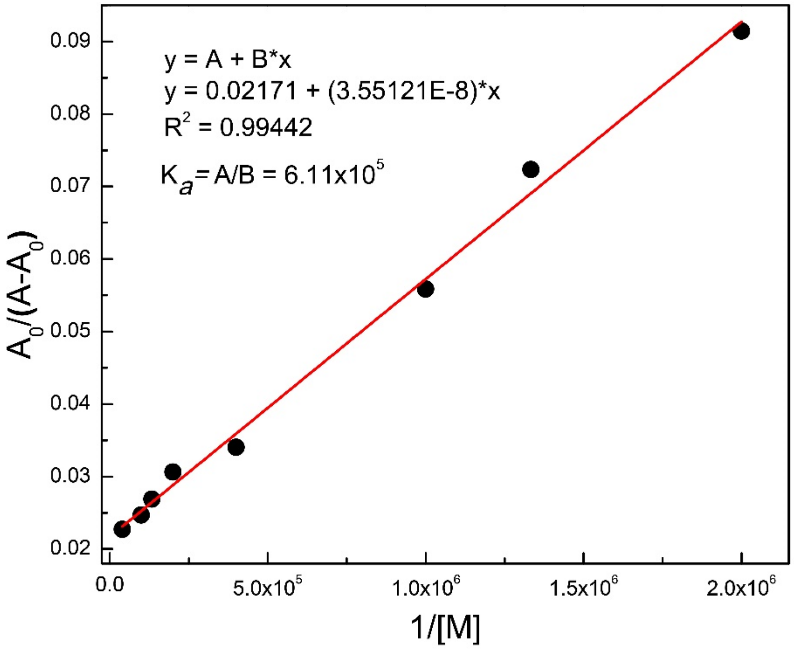

Figure S10. Linear regression curve of FH@Ni(MOF) obtained by plotting absorbance $A_0/(A-A_0)$ as a function of $1/[Hg^{2+}]$ in aqueous system.

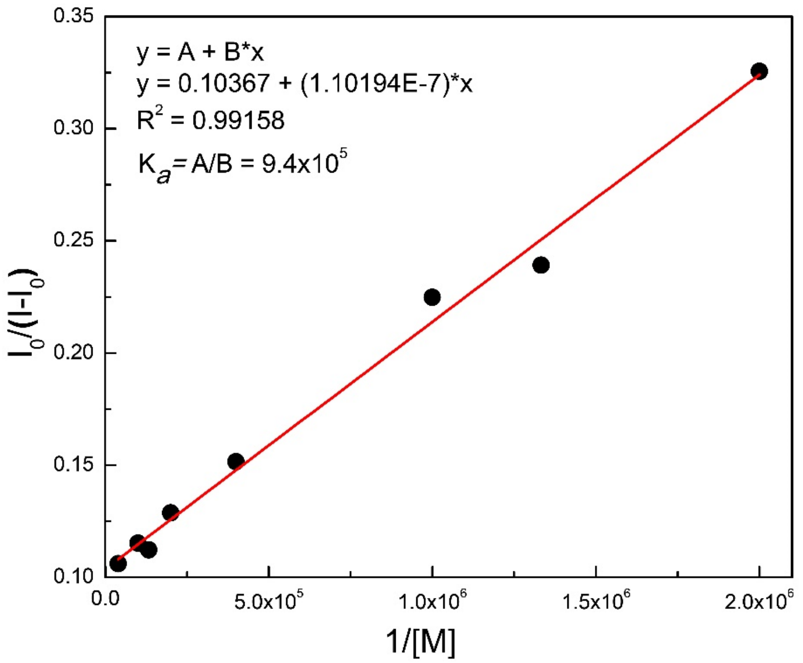

Figure S11. Linear regression curve of FH@Ni(MOF) obtained by plotting emission $I_0/(I-I_0)$ as a function of $1/[Hg^{2+}]$ in aqueous system. (λ_{ex} = 460 nm)

Section S4: **Determination of the detection limit**[2]

The detection limit was calculated based on the fluorescence quenching titration experiments. The fluorescence emission spectrum of FH@Ni(MOF) was measured five times from which the standard deviation of the blank measurement was achieved. To gain the slope, the fluorescence intensity at 523 nm was plotted as a concentration of the analytes. So the detection limit was calculated with the following equation:

Detection limit = $3\sigma/k$

Where σ is the standard deviation of blank measurement, k is the slope between the normalized fluorescence intensity versus analytes concentrations.

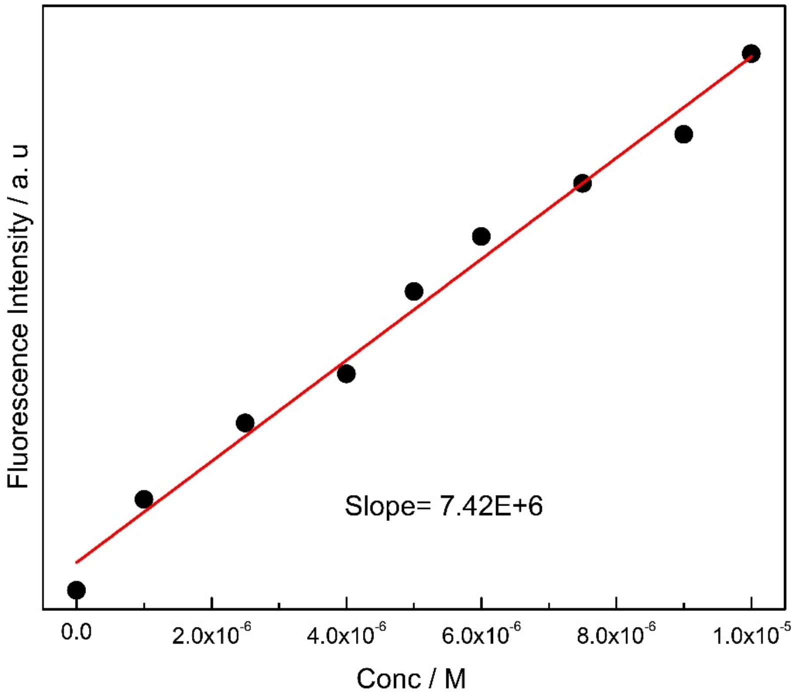

Figure S12. Linear region of fluorescence intensity (λ_{ex} = 460 nm and λ_{em} = 523 nm) for FH@Ni(MOF) suspensions in water upon incremental addition of Hg^{2+} solutions.

	Blank Readings	Hg^{2+}
1	Fluorescence Intensity	14.2862
2	Fluorescence Intensity	14.1745
3	Fluorescence Intensity	14.2356
4	Fluorescence Intensity	14.3421
5	Fluorescence Intensity	14.2928
6	Fluorescence Intensity	14.3143
Standard Deviation (σ)		0.06023
Slope (m)		7.42E+6
Detection limit (3σ/m)		0.024 μM / 5.0 ppb

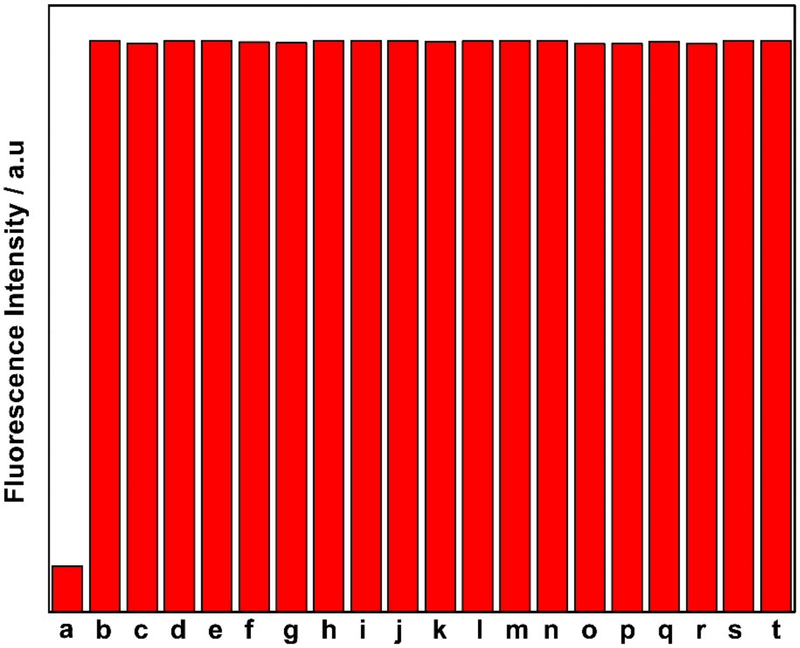

Figure S13. Competitive metal ion selectivity of FH@Ni(MOF): Bars indicate the fluorescence intensity (460 nm excitation, 523 nm emission). Salts of various metal ions (10^{-2} M) were added to FH@Ni(MOF) and Hg^{2+} (10^{-2} M) (a) FH@Ni(MOF) only, (b) $Ag^{+} + Hg^{2+}$, (c) $Pb^{2+} + Hg^{2+}$, (d) $Zn^{2+} + Hg^{2+}$, (e) $Mg^{2+} + Hg^{2+}$, (f) $Fe^{3+} + Hg^{2+}$, (g) $K^{+} + Hg^{2+}$, (h) $Co^{2+} + Hg^{2+}$, (i) $Al^{3+} + Hg^{2+}$, (j) $Fe^{2+} + Hg^{2+}$, (k) $Na^{+} + Hg^{2+}$, (l) $Cd^{2+} + Hg^{2+}$, (m) $Sr^{2+} + Hg^{2+}$, (n) $Rb^{+} + Hg^{2+}$, (o) Pd^{2+}+ Hg^{2+} (p) $Ni^{2+} + Hg^{2+}$, (q) $Fe^{3+} + Hg^{2+}$, (r) $Ga^{3+} + Hg^{2+}$, (s) $Cs^{+} + Hg^{2+}$, (t) $Ca^{2+} + Hg^{2+}$ in water.

Section S5: **Comparison of Absorbance of FH, Ni(MOF), and FH@Ni(MOF) with metal ions**

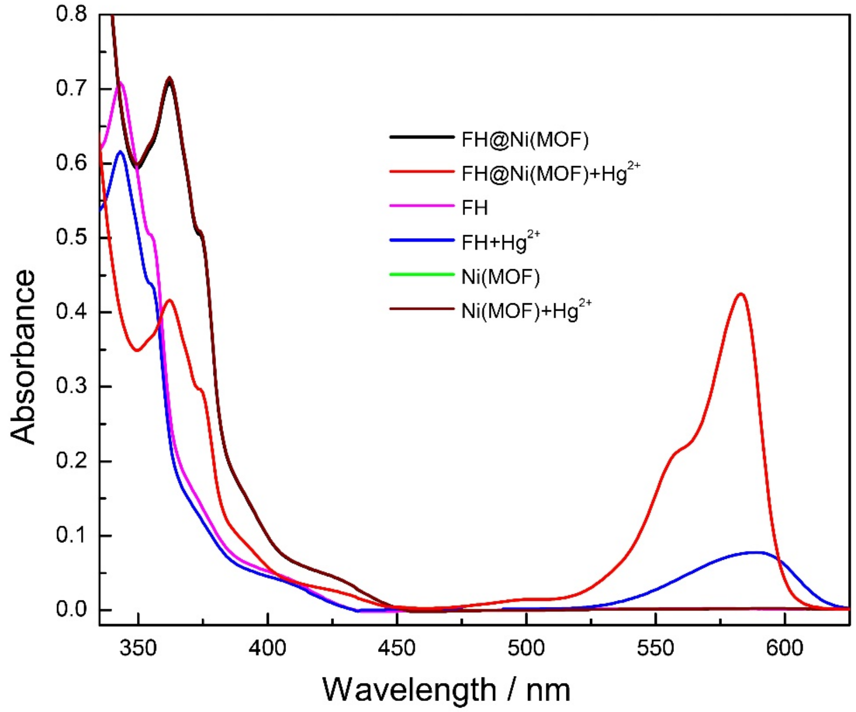

Figure S14. Change in the UV-vis spectrum of FH@Ni(MOF), FH, and Ni(MOF) in water upon addition of Hg^{2+} (10^{-2} M).

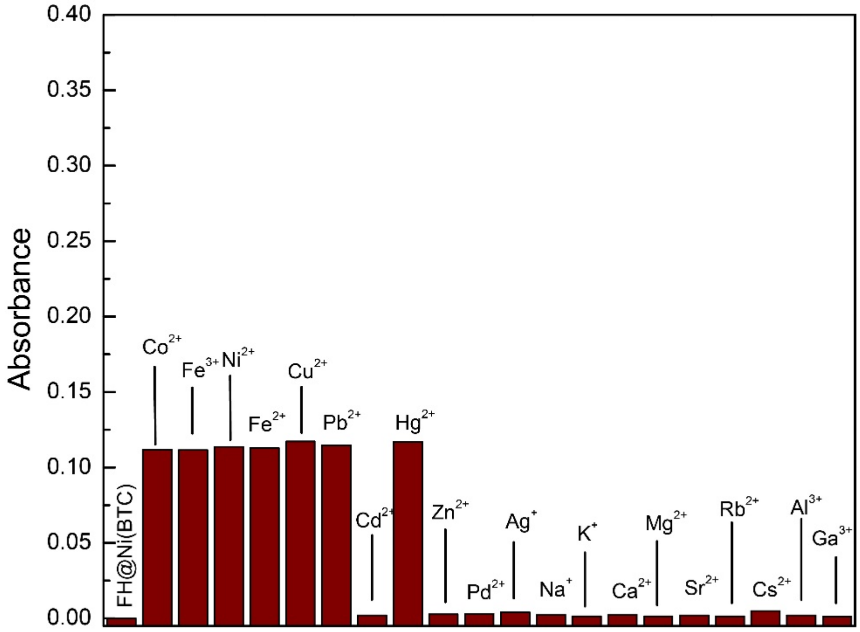

Figure S15. Change in the UV-vis absorbance of FH in water upon addition of 200 μL of different metal cations (10^{-2} M).

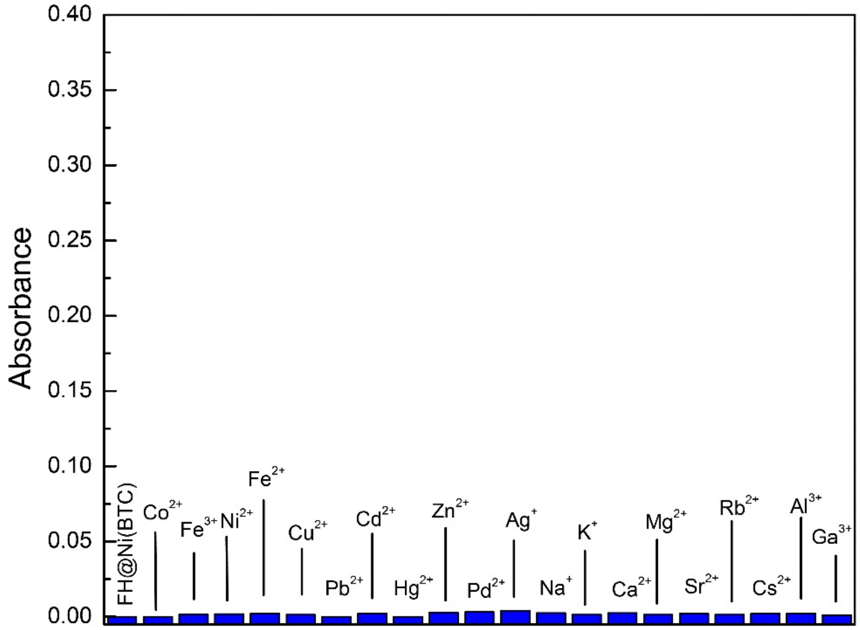

Figure S16. Change in the UV-vis absorbance of Ni(MOF) in water upon addition of 200 μL of different metal cations (10^{-2} M).

Section S6: **Recyclability of FH@Ni(MOF)**

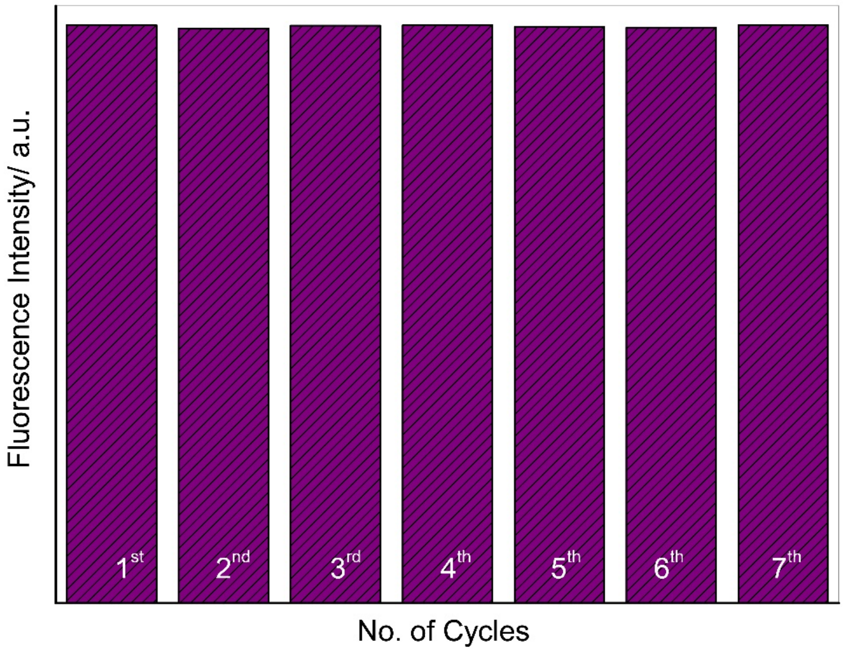

Figure S17. Bar diagram depicting the recyclability of FH@Ni(MOF) on addition of the Hg^{2+} up to 7 cycles.

Section S7: References

1. S. Fery-Forgues, M.-T. Le Bris, J.-P. Guett, B. Valeur, *J. Phys. Chem.* **1988**, *92*, 6233-6237.

2. Lv, R.; Wang, J.; Zhang, Y.; Li, H.; Yang, L.; Liao, S.; Gu, W.; Liu, X. *J. Mater. Chem. A* **2016**, *4*, 15494-15500.
